# Supplementary material for: Emissions of HFC-23 do not reflect commitments made under the Kigali Amendment
Source: Commun Earth Environ. 2024 Dec 21;5(1):783. doi: 10.1038/s43247-024-01946-y (PMC11663144; doi:10.1038/s43247-024-01946-y)
Supplement: Supplementary file 1 — Supplementary Information [file 43247_2024_1946_MOESM1_ESM.pdf]

Emissions of HFC-23 do not reflect commitments made under the Kigali Amendment

Supplementary Material

Ben Adam[1]*, Luke M. Western[1], Jens Mühle[2], Haklim Choi[3], Paul B. Krummel[4], Simon O'Doherty[1], Dickon Young[1], Kieran M. Stanley[1], Paul J. Fraser[4], Christina M. Harth[2], Peter K. Salameh[2], Ray F. Weiss[2], Ronald G. Prinn[5], Jooil Kim[2], Hyeri Park[6], Sunyoung Park[3,6] and Matt Rigby[1, 5]*

1. School of Chemistry, University of Bristol, Bristol, UK
2. Scripps Institution of Oceanography, University of California San Diego, La Jolla, California, USA
3. Kyungpook Institute of Oceanography, Kyungpook National University, Daegu, Republic of Korea
4. CSIRO Environment, Aspendale, Victoria, Australia
5. Center for Global Change Science, Massachusetts Institute of Technology, Cambridge, Massachusetts, USA
6. Department of Oceanography, Kyungpook National University, Daegu, Republic of Korea

Supplementary Tables

Year	Global annual emissions / Gg yr^{-1}	Global annual emissions 1-sigma / Gg yr^{-1}
1975	3.31	0.98
1976	3.41	1.19
1977	3.65	1.17
1978	4.06	1.05
1979	4.22	0.96
1980	4.33	0.94
1981	4.23	0.90
1982	3.83	0.91
1983	4.66	0.96
1984	5.09	0.87
1985	5.35	0.85
1986	5.75	0.86
1987	5.79	0.83
1988	6.75	0.83
1989	7.62	0.86
1990	7.08	0.83
1991	6.62	0.85
1992	7.28	0.82
1993	8.19	0.80
1994	8.18	0.81
1995	9.09	0.81
1996	9.52	0.83
1997	10.10	0.83
1998	10.42	0.77
1999	10.35	0.81
2000	11.06	0.89
2001	8.94	0.86
2002	9.47	0.86
2003	9.64	0.86
2004	11.87	0.88
2005	12.65	0.87
2006	14.34	0.89
2007	11.61	0.84
2008	11.46	0.63
2009	9.60	0.56
2010	10.53	0.60
2011	11.73	0.61
2012	12.92	0.63
2013	13.92	0.65
2014	14.44	0.69
2015	12.87	0.64

2016	12.79	0.66
2017	15.12	0.72
2018	16.83	0.73
2019	17.31	0.76
2020	16.51	0.76
2021	15.53	0.76
2022	13.95	0.77
2023	14.05	0.95

Supplementary Table 1: Global Emissions of HFC-23, derived from AGAGE mole fraction observations and the AGAGE 12-box model.

Year	Mean annual emissions / Gg yr^{-1}				Mean annual emissions 1-sigma / Gg yr^{-1}			
	Eastern China	North Korea	South Korea	Western Japan	Eastern China	North Korea	South Korea	Western Japan
2008	5.48	0.00	0.06	0.03	0.87	0.00	0.01	0.03
2009	5.47	0.00	0.08	0.03	0.79	0.00	0.01	0.02
2010	5.1	0.00	0.14	0.03	0.65	0.00	0.02	0.02
2011	4.38	0.00	0.10	0.03	0.60	0.00	0.01	0.01
2012	5.12	0.00	0.07	0.03	0.70	0.00	0.01	0.01
2013	6.81	0.00	0.11	0.05	0.96	0.00	0.03	0.03
2014	6.72	0.00	0.22	0.06	0.91	0.00	0.02	0.04
2015	5.75	0.00	0.30	0.07	0.63	0.00	0.02	0.04
2016	6.33	0.00	0.26	0.07	0.77	0.00	0.02	0.05
2017	7.35	0.00	0.22	0.10	0.92	0.01	0.03	0.07
2018	8.03	0.00	0.34	0.15	0.96	0.00	0.02	0.12
2019	7.98	0.00	0.35	0.14	1.12	0.00	0.03	0.12
2020	6.02	0.01	0.33	0.15	0.70	0.00	0.03	0.12
2021	4.63	0.01	0.38	0.17	0.54	0.00	0.03	0.14
2022	4.9	0.01	0.40	0.16	0.73	0.00	0.04	0.13
2023	5.61	0.01	0.23	0.10	0.67	0.01	0.02	0.07

Supplementary Table 2: Emissions of HFC-23 from eastern Asia for 2008-2023, aggregated by country, as derived in the FLEXINVERT+ Bayesian Inversion Framework

Year	Reported emissions from HCFC-22 manufacture / Gg	Total reported emissions / Gg
1990	9.42	9.35
1991	9.04	8.97
1992	9.19	9.12
1993	8.33	8.23
1994	8.43	8.39
1995	8.99	8.97
1996	8.88	8.92
1997	9.24	9.30
1998	10.03	10.10
1999	8.26	8.34
2000	7.70	7.77
2001	5.58	5.65
2002	4.63	4.71
2003	3.42	3.51
2004	3.42	3.52
2005	3.29	3.41
2006	2.63	2.76
2007	2.74	2.85
2008	2.47	2.58
2009	1.24	1.35
2010	1.44	1.56
2011	1.13	1.25
2012	1.19	1.32
2013	1.27	1.39
2014	1.41	1.54
2015	1.13	1.26
2016	1.02	1.14
2017	1.83	1.94
2018	2.15	2.26
2019	1.56	1.66
2020	1.50	1.59
2021	1.40	1.50

Supplementary Table 3: Annual emissions of HFC-23 for 1990-2021 reported by Annex I Parties to the United Nations Framework Convention on Climate Change (UNFCCC) in the 2023 National Inventory Reports (NIRs). These are totalled across all Parties, and both total emissions and those from HCFC-22 production alone are shown. Taken from NIR Submissions 2023 (https://unfccc.int/ghg-inventories-annex-i-parties/2023).

Country	Emissions / Gg				
	**2019**	**2020**	**2021**	**2022**	**2023**
Democratic People's Republic of Korea	0.0091	0.0091	0.0084	0.0084	None*
France	0.0045	0.0034	0.0003	0.0003	0.0003
Germany	0.0000	0.0000	0.0000	0.0000	0.0000
Japan	0.0000	0.0093	0.0086	0.0000	0.0000
Mexico	0.1119	0.0393	0.1285	0.0319	0.0000
Netherlands (Kingdom of the)	0.0184	0.0066	0.0174	0.0000	0.0047
Argentina	None	0.0362	0.0333	0.0173	0.0000
China	None	0.0481	1.0899	0.6374	None*
India	None	None	0.0000	0.0000	0.0000
Russian Federation	None	None	0.0000	0.0000	0.0000
Italy	None	None	None	0.0005	0.0003

Supplementary Table 4: Annual Emissions of HFC-23 from production of HCFC-22 reported to the United Nations Environment Programme under Article 7 of the Kigali Amendment to the Montreal Protocol. Data taken from the Ozone Secretariat (https://ozone.unep.org/hfc-23-emissions). * indicates no data had been published as of 13th November 2024.

Supplementary Figures

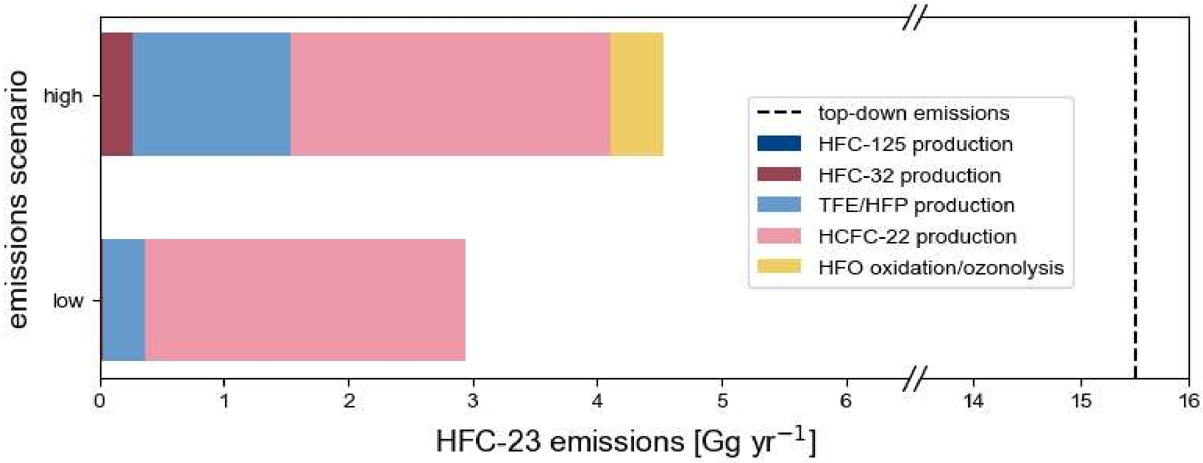

Supplementary Figure 1: Bottom-up inventory of HFC-23 emissions for 2021 under 'low' and 'high' emissions scenarios. Emissions from HFC-125 (dark blue) and HFC-32 (maroon) production are calculated using HFC production data[1] (see Methods), and the largest and smallest emissions factor from the range given by UNEP[2]. Emissions from TFE/HFP production (light blue) are estimated using HCFC-22 feedstock usage from ref.[3] and reports to UNEP[4,5], combined with the emissions factors range compiled by UNEP[4]. The emissions from HCFC-22 production (pink) are taken from the reports to UNEP[6] and the UNFCCC for 2021[7], as with the data in Figure 1. The contribution from the atmospheric oxidation of fluorinated gases (yellow) to the 'high' emissions scenario is taken from previous modelling work[8], and contributes 0.43 Gg yr^{-1}, although this is reported as an upper bound and the true contribution is likely to be lower (see Methods). In the absence of a lower bound in that study, atmospheric oxidation and ozonolysis has been excluded from the 'low' emissions scenario. This estimate is based on measurements of fluorinated gases in the atmosphere for 2020-2023, so 2021 is chosen as a representative year.

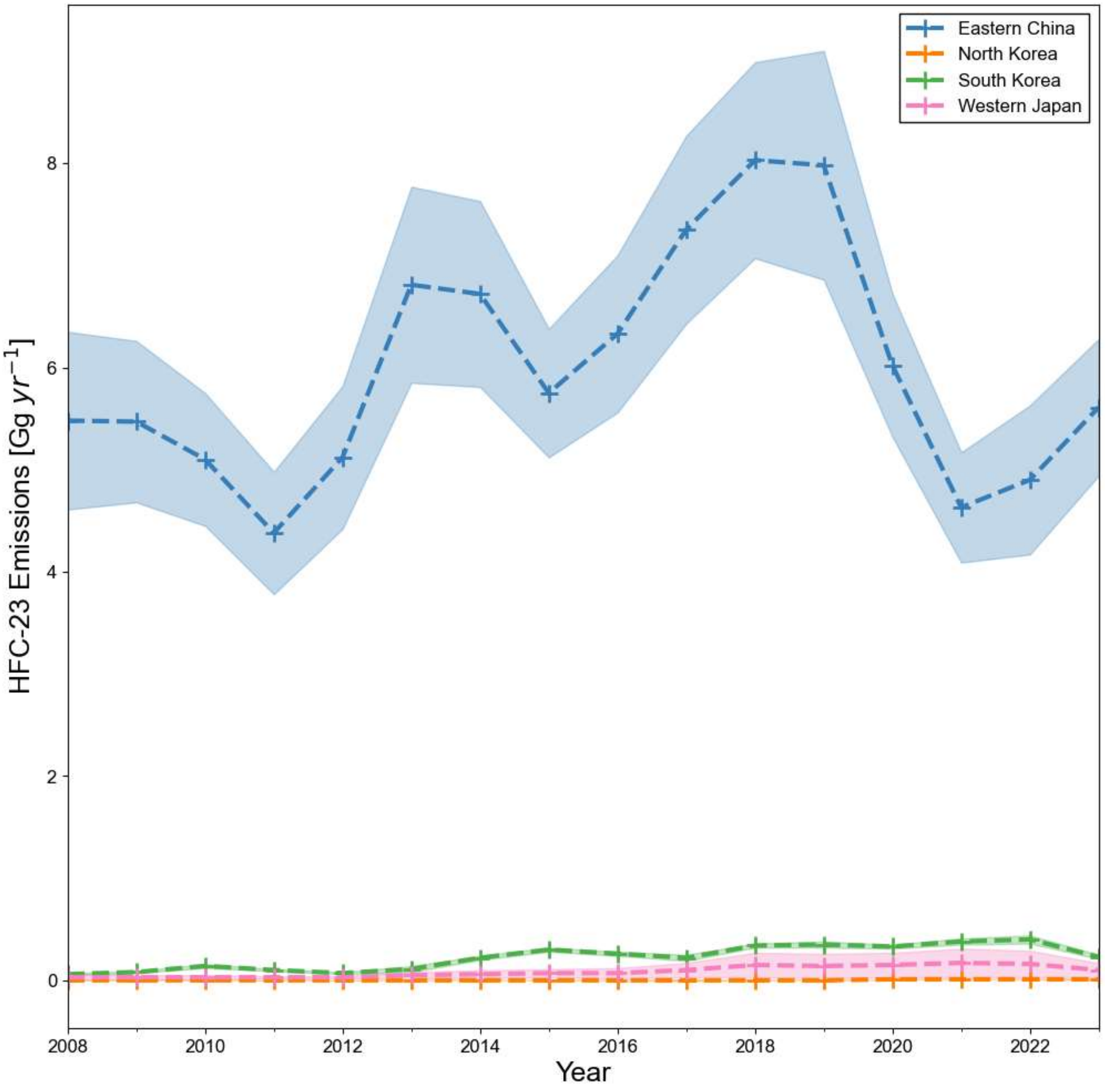

Supplementary Figure 2: Annual estimates of emissions of HFC-23 for eastern China (blue dashed line, defined as the provinces of Anhui, Beijing, Hebei, Jiangsu, Liaoning, Shandong, Shanghai, Tianjin and Zhejiang), North Korea (orange dashed line), South Korea (green dashed line) and western Japan (pink dashed line, defined as the provinces of Chūgoku, Kansai, Kyūshū, and Okinawa and Shikoku). The shading indicates the 1-sigma uncertainty in emissions for each year, as discussed in the Methods section.

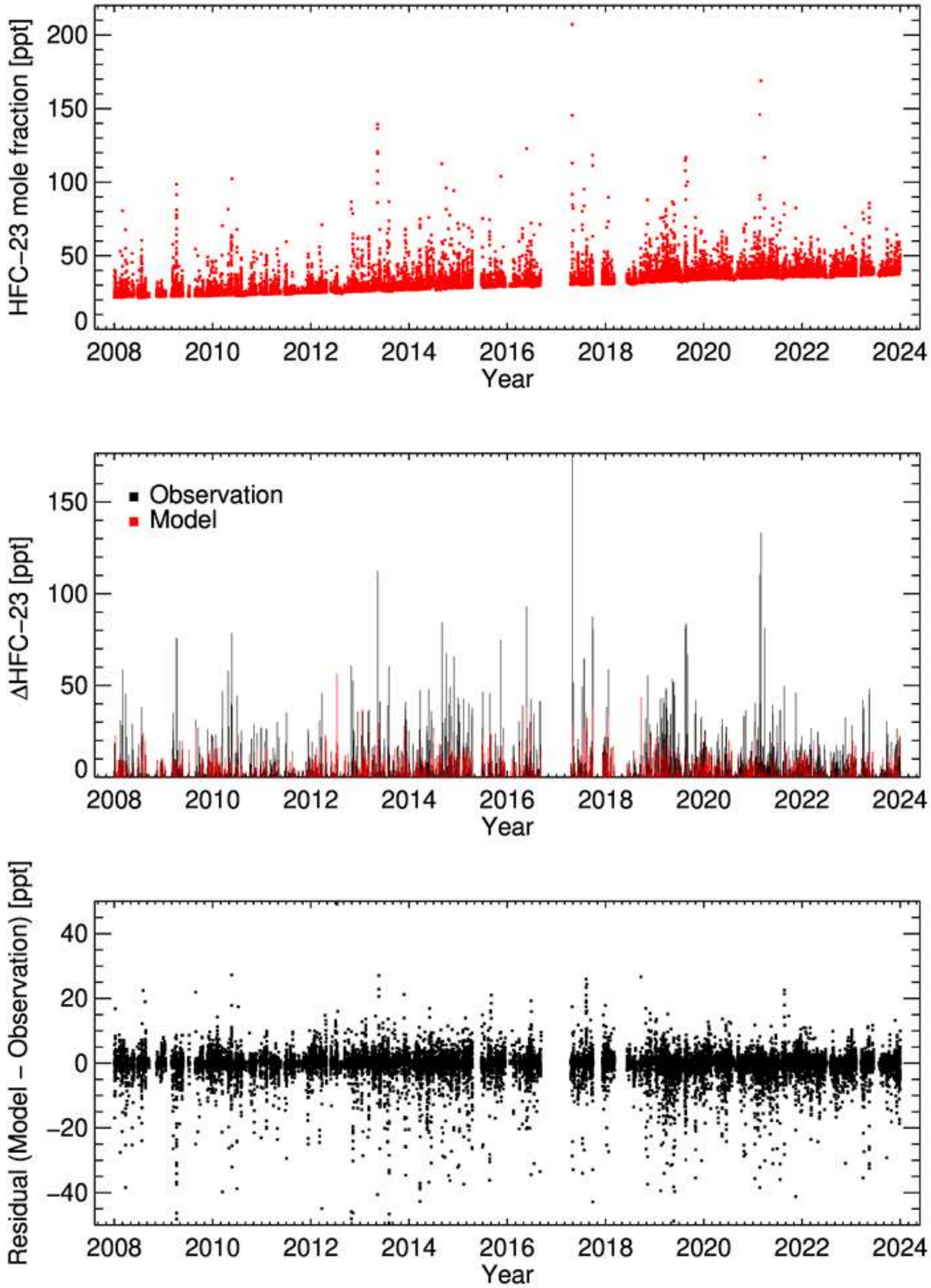

Supplementary Figure 3: A plot showing HFC-23 observations at the AGAGE station at Gosan, South Korea (33.3°N, 126.2°E) for the period 2008-2023 (top), the observed and modelled HFC-23 mole fraction enhancements above the rolling baseline at Gosan calculated using the AGAGE statistical pollution filtering algorithm[9] (middle) and the difference between these modelled and observed enhancements (bottom). Missing data are due to instrumental downtime.

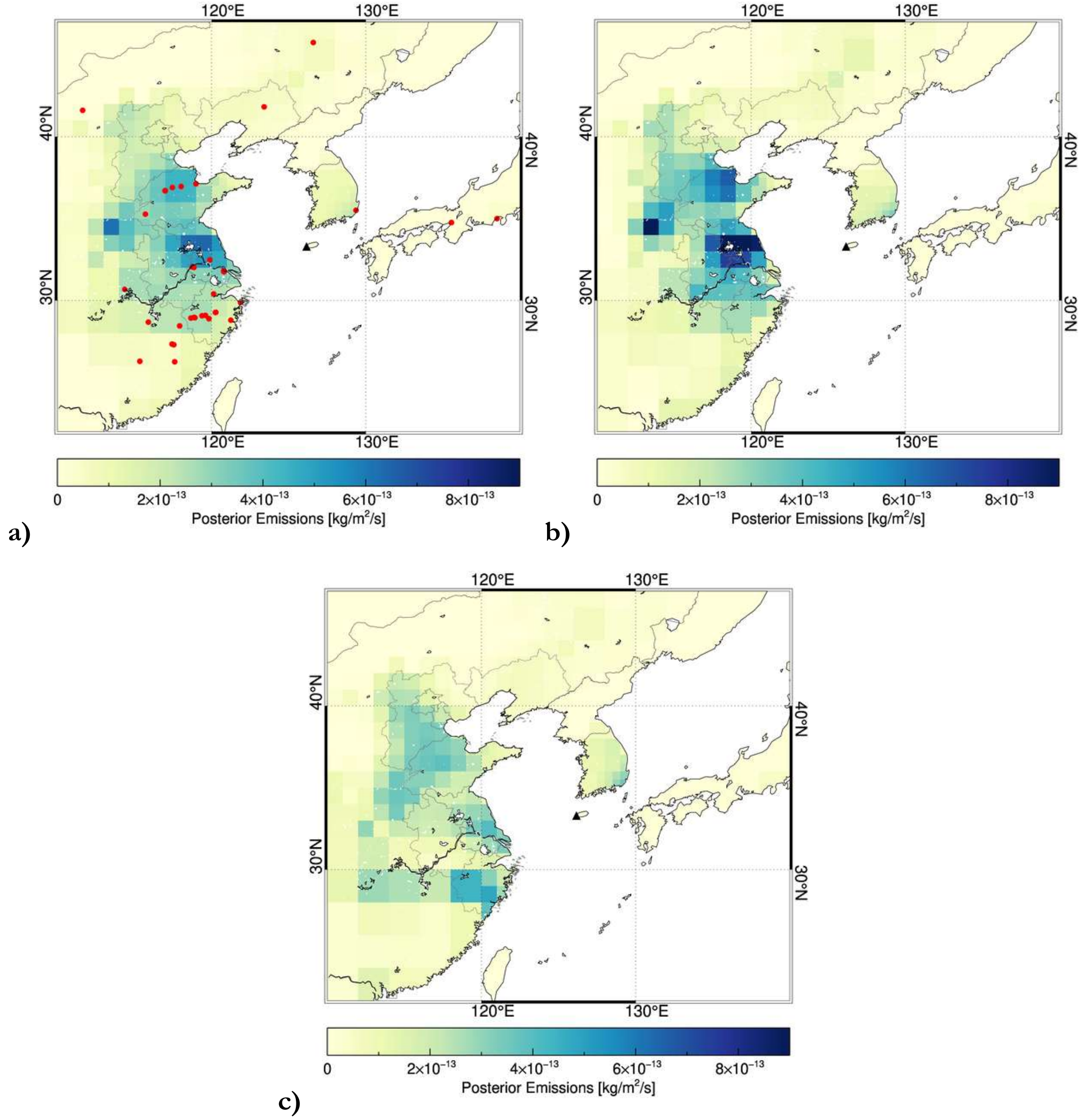

Supplementary Figure 4: Maps of time-averaged posterior mean emissions fluxes from east Asia from the FLEXINVERT+ inversion, covering a) the entire period 2008-2023, b) 2015-2019, corresponding to the first five years of China's hydrochlorofluorocarbon production phase-out management plan (HPPMP) and c) 2021-2023, corresponding to the implementation of the Kigali Amendment in China. The black triangle indicates the location of the Gosan station. In a), the locations of known HCFC-22 production facilities (taken from Park et al.[10]) are also shown with red circles. The dates of operation of these facilities are not all known, so they are not shown in b) and c). The spatial distribution of emissions correlates reasonably well with the locations of known HCFC-22 production facilities over the entire timeframe, although these plants are often co-located with other fluorochemical production facilities[3] and HFC emissions[11] and so these emissions cannot be attributed to HCFC-22 production on this basis.

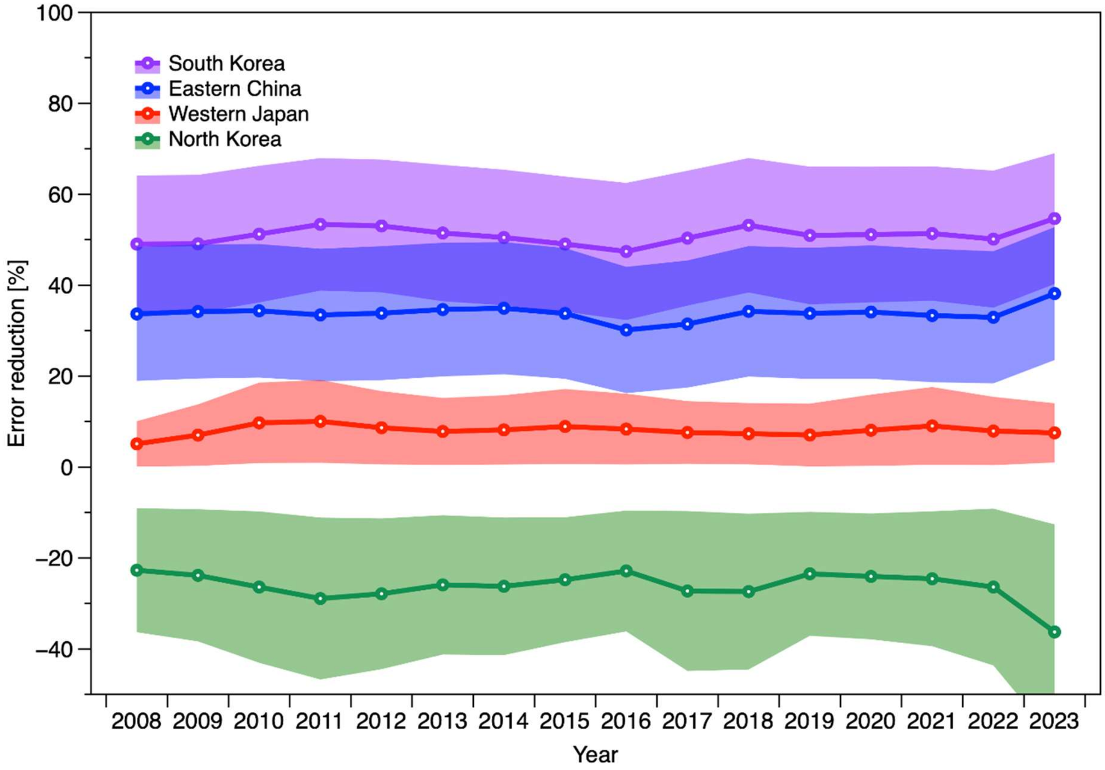

Supplementary Figure 5: A plot showing the uncertainty reduction between the prior and posterior HFC-23 emissions for eastern China (blue), western Japan (red), North Korea (green) and South Korea (purple). For each inversion, this is defined as the percentage reduction (between the prior and posterior) in the sum of the 1-sigma uncertainties in the grid cells corresponding to the given region. Positive values indicate the posterior error was lower than that in the prior. These are averaged across the 36 ensemble members, and the mean and standard deviation of these are shown here. Typical error reduction across the entire domain was approximately 30-40%.

Supplementary Methods

Regional Inverse Modelling Setup

An ensemble of 36 model runs was defined by the combination of three *a priori* spatial distributions of emissions, three scaling factors and four flux error magnitudes. The prior spatial distributions were 'Population', 'Asiaflat' and 'EastAsiaflat', as in ref.[10]. The 'Population' prior distribution has the national emissions (taken from ref.[12]) scaled by a global population density estimate[13]. The 'Asiaflat' prior has these same emissions spread evenly across the entirety of each country (North Korea, South Korea, China and Japan), allocating large prior emissions to regions to which the measurements are not very sensitive, such as western China and eastern Japan. The 'EastAsiaflat' prior has the national emissions spread evenly across only the regions within each country to which the measurements are highly sensitive. Such regions are defined as those where the variable grid resolution is 1°x1° or less. In this case, emissions were flattened across the entirety of North Korea and South Korea, due to these countries lying completely within the high sensitivity region. However, emissions were flattened only across eastern China and western Japan in this setup, and these regions are geographically defined in the Methods section of the main text. Three scaling factors (0.5x, 1.0x, 2.0x) were applied to the mean prior emissions, and four flux error magnitudes (50%, 100%, 200%, 300%) were applied to each variable resolution grid cell in the FLEXINVERT+ framework.

Supplementary References

1. Velders, G. J. M. et al. Projections of hydrofluorocarbon (HFC) emissions and the resulting global warming based on recent trends in observed abundances and current policies. Atmospheric Chemistry and Physics 22, 6087–6101 (2022).
2. United Nations Environment Programme. Report of the Technology and Economic Assessment Panel, September 2023. Volume 6: Response to Decision XXXIV/7: Strengthening Institutional Processes with Respect to Information on HFC-23 By-Product Emissions. https://ozone.unep.org/system/files/documents/TEAP-MCTOC-response-to-decision%20XXXIV7-report-september2023.pdf (2023).
3. Mühle, J. et al. Global emissions of perfluorocyclobutane (PFC-318, c-C4F8) resulting from the use of hydrochlorofluorocarbon-22 (HCFC-22) feedstock to produce polytetrafluoroethylene (PTFE) and related fluorochemicals. Atmospheric Chemistry and Physics 22, 3371–3378 (2022).

4. United Nations Environment Programme. Medical and Chemical Technical Options Committee: 2022 Assessment Report. https://ozone.unep.org/system/files/documents/MCTOC-Assessment-Report-2022.pdf (2022).
5. United Nations Environment Programme. Report of the Technology and Economic Assessment Panel, May 2023. Volume 1: Progress Report. (2023).
6. United Nations Environment Programme. HFC-23 emissions | Ozone Secretariat. https://ozone.unep.org/hfc-23-emissions.
7. United Nations Framework Convention on Climate Change. National Inventory Submissions 2023. UNFCCC https://unfccc.int/ghg-inventories-annex-i-parties/2023 (2023).
8. Montzka, S. A. et al. Report of the Scientific Assessment Panel, September 2024. Response to Decision XXXV/7: Emissions of HFC-23. https://ozone.unep.org/system/files/documents/SAP_Report_on_HFC23_September 2024.pdf (2024).
9. O'Doherty, S. et al. In situ chloroform measurements at Advanced Global Atmospheric Gases Experiment atmospheric research stations from 1994 to 1998. Journal of Geophysical Research: Atmospheres 106, 20429–20444 (2001).
10. Park, H. et al. A rise in HFC-23 emissions from eastern Asia since 2015. Atmospheric Chemistry and Physics 23, 9401–9411 (2023).
11. Choi, H. et al. Revealing the significant acceleration of hydrofluorocarbon (HFC) emissions in eastern Asia through long-term atmospheric observations. Atmospheric Chemistry and Physics 24, 7309–7330 (2024).
12. Stohl, A. et al. Hydrochlorofluorocarbon and hydrofluorocarbon emissions in East Asia determined by inverse modeling. Atmospheric Chemistry and Physics 10, 3545–3560 (2010).
13. Warszawski, L. et al. Center for International Earth Science Information Network—CIESIN—Columbia University. (2016). Gridded population of 200 the World, Version 4 (GPWv4): Population density. Palisades. NY: NASA Socioeconomic Data and Applications Center (SEDAC). Atlas of Environmental Risks Facing China Under Climate Change, 228, 2017.
